# Angiotensin II Receptor Blockers Reduce Tau/Aß42 Ratio: A Cerebrospinal Fluid Biomarkers’ Case-Control Study

**DOI:** 10.3390/pharmaceutics15030924

**Published:** 2023-03-12

**Authors:** Gemma García-Lluch, Carmen Peña-Bautista, Lucrecia Moreno Royo, Miguel Baquero, Antonio José Cañada-Martínez, Consuelo Cháfer-Pericás

**Affiliations:** 1 Research Group in Alzheimer Disease, Instituto de Investigación Sanitaria La Fe, 46026 Valencia, Spain; 2 Cátedra DeCo MICOF-CEU UCH, Universidad Cardenal Herrera-CEU, 46115 Valencia, Spain; 3 Department of Pharmacy, Universidad Cardenal Herrera-CEU, CEU Universities, 46115 Valencia, Spain; 4 Neurology Unit, Hospital Universitari i Politècnic La Fe, 46026 Valencia, Spain; 5 Data Science and Biostatistics Unit, Health Research Institute La Fe, 46026 Valencia, Spain

**Keywords:** Alzheimer’s disease, antihypertensives, amyloid, tau, angiotensin-converting enzyme inhibitor, angiotensin II receptor blockers, therapeutic strategies, personalized medicine

## Abstract

(1) Background: The role of antihypertensives in Alzheimer’s Disease (AD) prevention is controversial. This case-control study aims to assess whether antihypertensive medication has a protective role by studying its association with amyloid and tau abnormal levels. Furthermore, it suggests a holistic view of the involved pathways between renin-angiotensin drugs and the tau/amyloidß42 ratio (tau/Aß42 ratio); (2) Methods: The medical records of the participant patients were reviewed, with a focus on prescribed antihypertensive drugs and clinical variables, such as arterial blood pressure. The Anatomical Therapeutic Chemical classification was used to classify each drug. The patients were divided into two groups: patients with AD diagnosis (cases) and cognitively healthy patients (control); (3) Results: Age and high systolic blood pressure are associated with a higher risk of developing AD. In addition, combinations of angiotensin II receptor blockers are associated with a 30% lower t-tau/Aß42 ratio than plain angiotensin-converting enzyme inhibitor consumption; (4) Conclusions: Angiotensin II receptor blockers may play a potential role in neuroprotection and AD prevention. Likewise, several mechanisms, such as the PI3K/Akt/GSK3ß or the ACE1/AngII/AT1R axis, may link cardiovascular pathologies and AD presence, making its modulation a pivotal point in AD prevention. The present work highlights the central pathways in which antihypertensives may affect the presence of pathological amyloid and tau hyperphosphorylation.

## 1. Introduction

Alzheimer’s Disease (AD) is associated with alterations in the amyloid beta peptide (Aß) and tau proteins, as well as changes in cholinergic function [1,2,3]. The main AD cerebrospinal fluid (CSF) biomarkers are amyloid -ß42- (Aß42), total tau (t-tau), hyperphosphorylated tau (p-tau), and the tau/Aß42 ratio [4]. First, CSF Aß42 levels decrease in the development of the disease [5], which represents a reduced clearance from the brain into the blood, resulting in a higher accumulation of Aß plaques in the brain [6]. Second, the tau protein stabilizes microtubules in normal conditions as a compensatory mechanism against oxidative stress and Aß toxicity, and GSK3ß regulates its phosphorylation [7]. Elevated CSF tau levels are associated with neurodegeneration and are statistically associated with the progression from mild cognitive impairment to AD, and p-tau elevation reflects the formation of neurofibrillary tangles in the brain [8]. 

Since AD has a long asymptomatic period, risk factors such as hypertension are involved in its progression [1,9,10]. Hypertension is associated with a doubling in the likelihood of developing AD [2,9,11,12], and this risk increases if hypertension persists over the years [13]. In addition, hypertension causes oxidative stress and endothelial dysfunction, leading to blood vessel atrophy, which becomes particularly important with aging and is associated with cognitive impairment and increased Aß deposition in the brain [12].

Antihypertensives are of interest in dementia prevention due to their cerebrovascular structure protection and other mechanisms besides blood pressure control [13,14,15,16]. Nevertheless, not all antihypertensives have the same influence on AD, as their mechanisms of action differ. Antiadrenergic agents, such as the α-1-adrenoceptor antagonists, decrease peripheric vascular resistance [17] and are associated with (Aß) modulation [15,18]. On the other hand, diuretics may minimize cerebrovascular events and act on Aß peptides [15]. Vasodilator drugs enhance nitric oxide (NO), the role of which in AD is controversial [19]. Otherwise, diosmin stands out among the vascular vasoprotective agents because it reduces Aß and p-tau formation in mouse models [20], and ß-blockers may have the same effect on AD hallmarks [15]. Finally, calcium channel blockers (CCB) are highlighted due to their neuroprotective properties [15,21,22,23,24] and, along with the renin-angiotensin system (RAS)-acting agents, they both appear to be the most effective option in AD risk modulation [16]. In this sense, the angiotensin-converting enzyme inhibitors (ACEi) and the angiotensin II receptor blockers (ARBs) stand out as the main drugs acting on the RAS system. They both are associated with AD risk reduction, but their mechanisms of action differ. While the ACEi prevent the inactivation of bradykinin and the formation of Angiotensin II (1–8), affecting AT1R and AT2R [25,26], ARBs are AT1 receptor antagonists, and, therefore, they enhance AT2, Ang IV, and Ang (1–7) receptors [3,25,26,27,28,29]. 

For all the above, the present study aims to evaluate the associations between antihypertensive treatments and AD CSF biomarkers. 

## 2. Materials and Methods

### 2.1. Participants and Study Design

The present work is a retrospective case-control study conducted at the Neurology Unit of the University and Polytechnic Hospital La Fe (Valencia, Spain). The Ethics Committee for Biomedical Research at CEU Cardenal Herrera University and the Medicaments Research Ethics Committee at the Health Research Institute Hospital La Fe have approved this study (CEI21/052 and 202-705-1). 

The participants were recruited through a medical interview between January 2017 and December 2020. The enrolled patients received information and signed the informed consent, following the Declaration of Helsinki, the Good Clinical Practices, and local regulations.

The inclusion criteria for this study were to be between 50 and 80 years old, sign the informed consent, and have medical records of CFS biomarkers (Aβ42, t-tau, p-tau), neuropsychological evaluation, and medication intake. The exclusion criteria for the present study were not to meet the inclusion criteria, be enrolled in a clinical trial, have other neurological diseases such as epilepsy, multiple sclerosis, or brain damage, or have psychiatric disorders, such as depression (major disorder) or bipolar disorder. In addition, patients with severe dementia or previous disabilities were excluded.

The patients’ diagnoses were based on The National Institute on Aging-Alzheimer’s Association clinical criteria [30]. Therefore, a neuropsychological evaluation based on the Clinical Dementia Rating (CDR) [31], the Repeatable Battery for the Assessment of Neuropsychological Status-Delayed Memory (RBANS.DM) [32], the Mini-Mental State Examination (MMSE) [33], the Functionality Assessment Questionnaire (FAQ) [34], and the AD Cooperative Study ADL Scale for Mild Cognitive Impairment (SDCS-ADL-MCI) [35] were performed. In addition, neuroimaging and CFS biomarkers (ß42, t-tau, p-tau, tau/Aß42 ratio) were assessed. Patients were "and tau/Aß42 ratio were found. Neuropsychological evaluation was considered to optimize a patient’s diagnosis and establish a patient’s cognitive decline stage. Patients with normal CSF levels and who were cognitively healthy were classified as control participants. All efforts were made to include a biologically defined control group (CSF biomarkers) in the study. 

### 2.2. Data Source and Variables

The patients were anonymized, and the electronic health system was used to perform an exhaustive review of their medical records at the Polytechnic University Hospital La Fe (Valencia). Thus, age, sex, smoking history, and comorbidities such as hypertension were registered. Furthermore, total and high-density lipoprotein (HDL) cholesterol, as well as blood pressure levels, were calculated from the average of two or more measurements, preferably within six months before or after diagnosis. Those variables were gathered at the participant’s hospital and related healthcare centers.

CSF samples were obtained as part of the diagnosis protocol at the Polytechnic University Hospital La Fe (Valencia). From 5 to 10 mL of CSF was collected and stored at −80 °C until analysis. Biochemical determinations (Aβ42, t-tau, p-tau) were carried out by a chemiluminescence immunoassay [36]. Specifically, the CSF biomarker cut-off established for t-tau/Aß42 was >0.51 and was >485, >56, and <725 pg/mL for t-tau, p-tau, and Aß42, respectively [37].

An antihypertensive treatment prescription was acquired by a medical history review, and it was registered by Yes/No using the Anatomical Therapeutical Chemical (ATC) code of the WHO Collaborating Centre for Drug Statistics Methodology (WHO) “https://www.whocc.no/atc_ddd_index/ (accessed on 1 May 2021)”. ATCs were firstly regrouped and analyzed by therapeutic subgroup (2nd ATC level) and, secondly, by introducing agents acting on the RAS as their pharmacological subgroup (3rd level). As for the C02 ATC group, only doxazosin was found. Thus, for greater clarity, reference to this group will be made directly to this active ingredient. The same situation was performed with vasoprotective medication, with calcium dobesilate as the representative drug. Finally, the duration of treatment was represented in months.

### 2.3. Statistical Analysis

The data were summarized using the median (1st and 3rd quartiles) for the numeric variables and the absolute frequency (%) for the qualitative variables. The biomarkers were log-transformed to avoid skewed data. 

On the one hand, logistic regression models were performed to assess the relationship between clinical classification attending to CSF biomarkers (AD group, control group) and age, gender, and systolic and diastolic blood pressure. In addition, 2-way interactions with systolic blood pressure (SBP), X “antihypertensives”, diastolic blood pressure (DBP) X “antihypertensives”, and hypertension X “antihypertensives” were explored. Finally, conditional effects with their 95% CI were depicted. 

On the other hand, elastic net linear regression models were adjusted for each biomarker (ß42 amyloid, t-tau, p-tau, and t-tau/ß42 ratio) to select their associated characteristics. The general model included the following variables: age, sex, SBP, DBP, diabetes mellitus type 2, total cholesterol, smoking habit, number of chronic treatments, and antihypertensive drugs intake (doxazosin, diuretics, peripheral vasodilators, calcium dobesilate, beta-blocking agents, calcium channel blockers, plain ACEI, combinations of ACEi, plain ARBs, and combinations of ARBs).

The elastic net regularization method of the estimated beta coefficients improves upon ordinary least squares. It linearly combines the L1 and L2 penalties of the lasso and ridge methods. The regularization parameter λ determines the amount of regularization. An optimal value for λ was determined by performing a 10-fold cross-validation, which yielded the minimum cross-validated mean-squared error (CVM). A median of 1000 repetitions of the cross-validation was calculated to improve lambda’s robustness. 

The ARBs and ACEi and their relation to t-tau/ß42 amyloid were analyzed by multivariable logistic regression. Multiple comparisons were performed to assess the differences in the before-mentioned groups. The goodness of fit for the adjusted model was carried out using simulated scale residual diagnostics.

All the statistical analyses were performed using R (V. 4.0.3.) and the packages glmnet (V.4.1-3), click (V.0.8.0), ggeffects (V.1.1.1), ggplot2 (V.3.3.5), and DHARMa (V. 0,4.4).

## 3. Results

### 3.1. Participants

**Figure 1:**
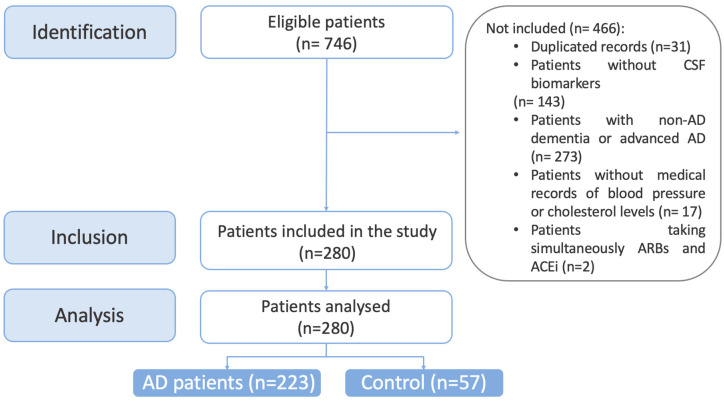
STROBE participant’s selection flow chart. ACEi= angiotensin-converting enzyme inhibitor; AD = Alzheimer’s Disease; ARBs = angiotensin II receptor blockers; CSF = cerebrospinal fluid.

Seven hundred and forty-six participants were enrolled in the present study. From these, duplicated records due to follow-up (*n* = 31), patients without CSF biomarkers (n = 143), those diagnosed with other dementias (non-AD), or those with moderate or severe dementia due to AD (*n*= 273) were not included. Finally, the patients without medical records of total cholesterol levels or blood pressure (*n* = 17) or with the simultaneous prescription of ARBs or ACEi (*n* = 2) were excluded (see Figure 1). 

From the initial cohort, 280 patients were included. They were classified as AD and cognitively healthy patients, according to their CSF levels. Thus, 57 participants were considered cognitively healthy patients (controls) and 223 were considered AD patients (cases), of whom 160 patients (71.75%) had mild cognitive impairment due to AD and 63 patients (28.25%) had mild dementia due to AD. 

### 3.2. Demographic and Clinical Data of Participants

Table 1 shows the demographic and clinical variables for each group of participants. As can be seen, the AD patients were older than the controls, were predominantly female, and had more chronic concomitant medications prescribed. 

Regarding cardiovascular risk factors, the patients with AD were predominantly non-smokers and had lower total cholesterol levels. In contrast, they had a greater rate of lipid-modifying prescription, SBP levels, and hypertension than the control patients. However, the control patients were more prone to taking antidiabetic drugs and having higher DBP than the case group (Table 1).

### 3.3. Hypertension and Alzheimer’s Disease

Multivariate logistic regression was performed. In addition, age, gender, and blood pressure levels were analyzed and compared to the presence of AD. SBP and antihypertensive prescription statistical interaction were explored without significant differences. Thus, the [SBP x antihypertensive intake] interaction was removed. 

A positive association was found between the likelihood of suffering from AD and age (OR = 1.174, IC95% [1.105; 1.255], *p*-value < 0.001) and higher SBP (OR = 1.036, IC95% [1.004; 1.071], *p*-value = 0.033). On the contrary, men seemed less likely to develop AD than women despite the result being non-significant (OR = 0.513, IC95% [0.246; 1.051], *p*-value = 0.07). No differences were found regarding diastolic blood pressure and antihypertensive intake (see Table S1 of Supplementary Information).

### 3.4. Antihypertensive Drugs and Alzheimer’s Disease Biomarkers

Each therapeutic subgroup prescription was examined to assess whether antihypertensive drugs are associated with AD (Table 2). As can be seen, the AD patients were older when the first antihypertensive drug was prescribed and took the medication for more years. In addition, ß -blocking agents and CCB were consumed more among the AD patients, whereas diuretics and agents acting on RAS were the most common drugs among the control patients.

Moreover, all the models associated older age with impaired CFS biomarkers levels (Table 3). Additionally, a trend was observed between antidiabetic consumption and higher Aß42 and lower t-tau/Aß42, whereas being male seemed to be linked to lower t-tau levels. CCB seemed to be associated with a higher t-tau/ß42 amyloid ratio. Finally, plain ACEi drugs were associated with higher t-tau and t-tau/ß42 amyloid levels, whereas combinations of ARBs were related to lower levels of this biomarker.

### 3.5. ACEi and ARBs Pharmacological Subgroups

Since ARBs and ACEi showed opposite t-tau/Aß42 ratio effects (Table 3), a deeper analysis was performed (Table 4). As a result, it was observed that a significant proportion of the patients with AD were taking ACEi, whereas ARBs were the most consumed drugs among the control patients. Moreover, almost all the control patients were taking plain ACEi. 

**Figure 2:**
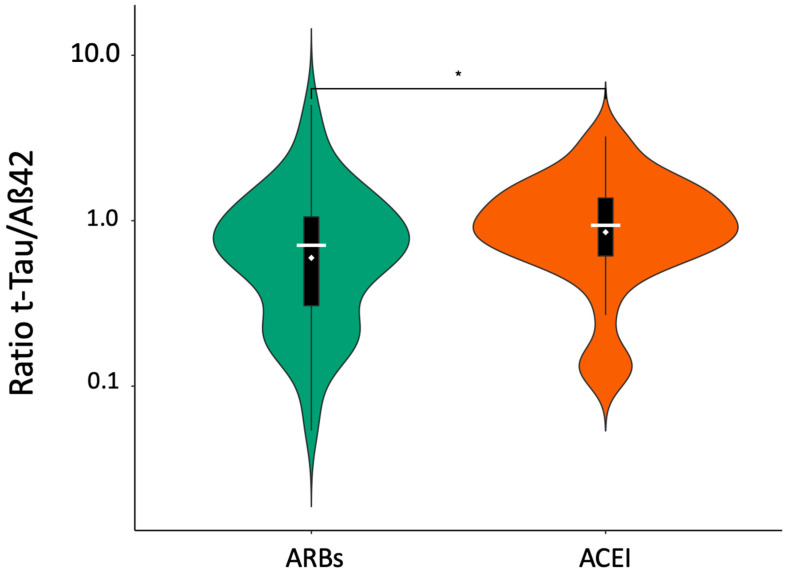
Comparison of ARBs and ACEi consumption with t-tau/Aß42 ratio values using violin model. * = Level of significance is provided by Wilcox Test. Aß42 = Amyloid beta 42; ACEi = Angiotensin-converting enzyme inhibitors; ARBs = Angiotensin receptor blockers; t-tau = total CSF tau levels; t-tau = total tau.

Firstly, it was observed that the consumption of ARBs was significantly associated with a lower t-tau/Aß42 ratio when compared to ACEi (see Figure 2).

Secondly, multivariable logistic regression was performed to confirm the abovementioned results and predict the t-tau/Aß42 ratio association with ARBs and ACEi (see Table S2). The model included sex and age as covariables because they seemed to be the variables with the strongest association with AD. It was observed that combinations of ARBs consumption were associated with a 30% lower t-tau/Aß42 ratio than plain ACEi consumption (estimate = −0.334, IC95%, [−0.613, −0.055], *p*-value = 0.019).

**Figure 3:**
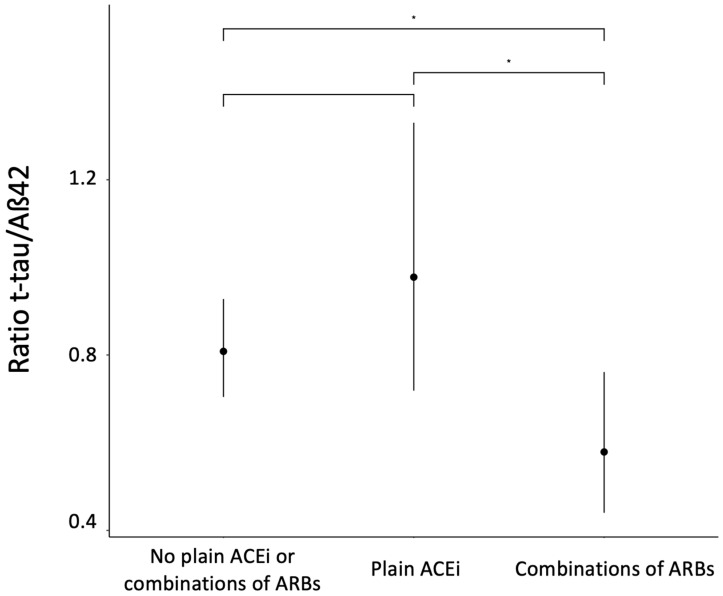
Conditional effects of t-tau/Aß42 ratio to combination ARBs and plain ACEi and Tukey multiples comparisons. Adjusted for age = 69 years, female. (*) = *p*-value <0.05. ß42 = Amyloid beta 42; ACEi = Angiotensin-converting enzyme inhibitors; ARBs = Angiotensin receptor blockers; t-tau = total tau.

Thirdly, statistical differences in the t-tau/Aß42 ratio between patients taking combinations of ARBs and patients consuming plain ACEi were observed (estimate = −0.5242, IC95% (−0.1984; −2.643), *p*-value = 0.026), as well as between patients taking combinations of ARBs and those not taking plain ACEi or combinations of ARBs (estimate = −0.3339, IC95% (0.1418; −2.354), *p*-value = 0.0485) (Figure 3).

## 4. Discussion

The present study compares the differences between the different antihypertensive treatments and the alteration of fluid biomarkers for AD. Previous studies point out that antihypertensive medication is associated with AD risk reduction, but they are mainly based on cognitive test evolution or dementia diagnosis conversion [22,38,39]. In order to follow a standardized biological criterion, CSF biomarkers were used in AD diagnosis. To our knowledge, this is one of the few antihypertensive studies in AD that defines case and control groups based on CSF biomarker levels. For instance, a clinical trial performed in 2012 about ACEi modulation of ACEs activity in CSF included fourteen volunteers [40]. Moreover, the study of Hestad and co-workers included eight patients with subjective memory complaints as a control group out of 72 subjects [8]. Finally, Nation et al. performed a study of antihypertensives based on CSF AD biomarkers in 2016, but it just included 124 patients [41].

This study shows that high SBP and AD are associated, which is consistent with the recent findings of Hestad et al. who found an association between SBP and CSF t-tau concentrations with lower delayed memory [8,41]. In addition, Affleck and co-workers showed in 2020 that the amyloid brain burden was lower in normotensive AD patients than in hypertense AD patients [2]. Hypertension seems to be associated with an increase in ß-secretase, the enzyme responsible for activating the amyloidogenic pathway of Aß production, and an increase in the Aß42/Aß40 ratio [12]. In addition, several studies affirm that the association between SBP and dementia is significant in midlife but not later life [13]. Altogether, it seems that vascular damage is associated with AD. Due to the long period that elapses from when the pathological pathways begin to be altered until the first symptoms appear, it is possible in middle age when this factor becomes especially important.

Furthermore, this study compares the association between antihypertensive use and abnormal AD CSF biomarkers. A previous study performed by Affleck and co-workers in 2020 revealed that patients who take this medication have lower neurofibrillary tangle formation [2]. Nevertheless, when Hestad et al. compared antihypertensive consumption and cognitive functions, they showed worse cognitive function in the antihypertensive consumption group [8]. 

We did not observe AD diagnosis or CSF biomarker differences in our group compared to antihypertensive consumption per se. Nevertheless, it was observed that the AD patients received their first antihypertensive drug at an older age and for more years in our cohort than the control patients. Therefore, antihypertensives may not avoid AD development, but they may affect mild cognitive impairment or progression by minimizing vascular damage at the early stages and through mechanisms other than blood pressure control [16]. As a result, each antihypertensive class was analyzed separately and compared in four AD-biomarker models.

First, doxazosin prescription was associated with higher CSF Aß42 concentrations and lower t-tau and a lower tau/Aß42 ratio. In addition, despite scarce literature about doxazosin and AD biomarkers, a recent study showed that doxazosin prevented Akt reduction, avoiding tau phosphorylation in an in vitro model of organotypic hippocampal cultures exposed to Aß [18]. However, our results must be taken carefully due to the reduced number of patients taking this drug in our cohort.

Regarding vasoprotective medication, calcium dobesilate releases NO, producing vasodilation [42,43]. The role of NO with biomarkers is controversial since it is involved in GSK-3ß activation and the consequent tau phosphorylation [19], as well as with Akt and cyclic-AMP-response-element-binding protein (CREB), which promotes cell survival and neuroprotection [44]. Recent studies indicate that an NO neuroprotective or neurotoxic effect depends on its concentration. It modulates heme-metals-Aß binding and plays a key role in Aß toxic effects [45]. In our cohort, calcium dobesilate seemed to be associated with higher CSF amyloid concentrations and a lower tau/Aß42 ratio. Nevertheless, only one patient was taking this antihypertensive in our sample, so further studies are needed to obtain conclusions.

As for beta-blocking agents, we did not observe any statistical difference between their consumption and AD hallmark alteration, which is consistent with previously published research [16]

In other matters, CCB highlights promising results in dementia prevention [15,22,23,24]. Intracellular calcium is elevated in elderly patients and plays a part in neurodegeneration, amyloid production enhancement, and tau hyperphosphorylation [15,16,23,46]. Thus, CCB may downregulate intracellular calcium levels and slow amyloid production [16,23]. In addition, CCB can enhance cerebral vascularization [15] and, in the case of nimodipine, can act as a cerebral vasodilator [46]. Among CCB, the dihydropyridine compounds stand out with promising results in Aß42 clearance [21,23]. Nevertheless, as shown in the work of Bachmeier et al., not all the dihydropyridines have the same effect on brain vasculature, and their effect on Aß42 clearance may not depend on blood–brain barrier (BBB) penetration. Whereas drugs such as nimodipine or nitrendipine are likely to enhance Aß clearance from the blood to the brain; others, such as amlodipine or nifedipine, do not seem to facilitate Aß42 transcytosis across the BBB in in vivo models, despite the fact that all of them can cross the BBB [21]. 

When we analyzed our cohort, we noticed that CCB was associated with a higher tau/Aß42 ratio and that the most CCB consumed was amlodipine. Moreover, just one patient took nimodipine when the lumbar puncture was performed. A recent study by Sadleir Id and colleagues aimed to explore whether nimodipine could modify amyloid pathogenesis when it begins in mouse models, but it did not show any changes in the Aß42 or total Aß levels nor amyloid plaque deposition [46]. In addition, it was shown in work performed by Murray and colleagues in 2002 that CCBs were not associated with dementia prevention. Most of the prescribed dihydropyridines in our study were the same that did not boost Aß42 clearance in a study performed by Bachmeier and colleagues in 2011 [21,39]. Moreover, in the Baltimore Longitudinal Study of Aging, CCB did not reduce the incidence risk of AD [47], an effect that was neither observed in the Gingko Evaluation of Memory Study [48] nor the NIVALD study, the phase III clinical trial that tested nivaldipine vs. placebo in AD patients [49].

Altogether, it could explain why we did not observe a protective effect in our sample, although further studies are needed to elucidate the exact mechanism by which amlodipine may increase the tau/Aß42 ratio.

Lastly, RAS drugs stand out among the antihypertensive drugs thanks to their potential ability to limit Aß plaques [14] and neurofibrillary tangle formation [2,14]. There is evidence of a dysregulation of endogenous RAS activity in AD patients, which has been confirmed in post-mortem brain tissue [14,27]. As recently reviewed by Gouveia and colleagues, ARBs and ACEi may be more effective at preventing AD than other antihypertensives [14,16,24,29]. Nevertheless, the bibliography suggests that certain ACEi are associated with the risk of dementia [12], whereas ARBs may act as neuroprotectors [16,29]. As a result, studying the effects of both drugs and their influence on CSF AD biomarkers is of interest. 

The ACEi mechanism of action prevents the formation of Angiotensin II (1–8) and the degradation of plasma bradykinin through ACE inhibition, thus, contributing to inflammation, vascular and blood–brain barrier permeability, and impaired cerebral flow [50,51,52,53,54]. 

Moreover, ACE1 degrades Aß-42 into Aß40, its soluble form [3,55], and studies show that ACEi can modify ACEs activity in CSF [40]. As a result, if ACE becomes blocked by ACEi, the clearance of Aß42 may not succeed, and plaques may accumulate in the brain [41].

Conversely, ACE inhibition may enhance the bradykinin concentrations in plasma and B1R and B2R activity in microglial cells [56]. B2R expresses constitutively under normal conditions, is activated in acute inflammation [51,53,57,58,59], and has a higher affinity for bradykinin and Lys-bradykinin peptides [53,58]. However, B1R is upregulated by chronic inflammation [53,58,60] and has a higher affinity for Lys-des-Arg9-BK and des-Arg9-BK [50,51,53,57,58,61]. Moreover, the B1R-derived pro-inflammatory cytokine release may contribute to BBB permeability and its disruption [58], being an essential pathophysiological mediator of cerebrovascular dysfunction, neuroinflammation, and Aß pathology in AD [62].

**Figure 4:**
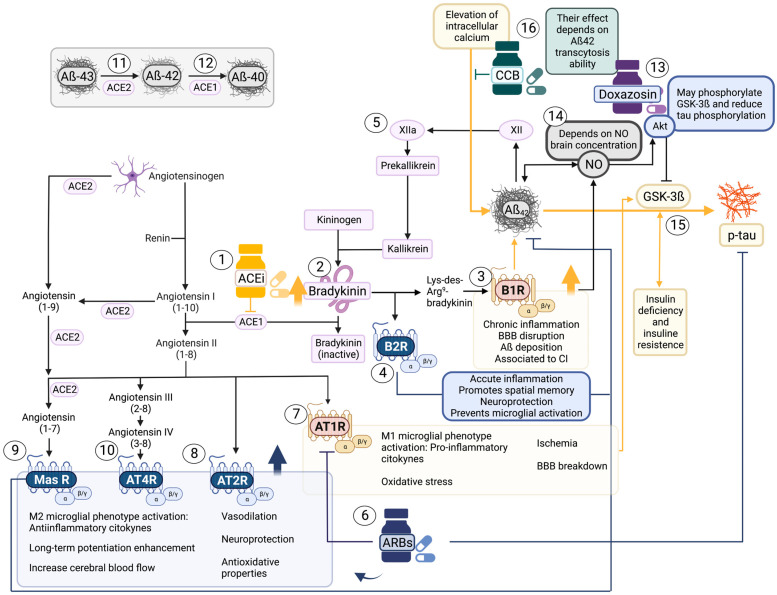
Proposed mechanism of action among antihypertensive drugs and CSF amyloid and tau alterations. Pathological processes are represented in yellow, whereas neuroprotective mechanisms appear in blue. Created with BioRender.com. Aß40 = Amyloid beta 40; Aß42 = Amyloid beta 42; ACEi = Angiotensin-converting enzyme inhibitors; ACE1 = Angiotensin-converting enzyme 1; ACE2 = Angiotensin-converting enzyme 2; Akt = Protein kinase B; ARBs = Angiotensin receptor blockers; AT1 = Angiotensin 1 Receptor; AT2 = Angiotensin 2 Receptor; AT4R = Angiotensin 4 Receptor; B1R = Bradykinin 1 receptor; B2R = Bradykinin 2 receptor; CCB = Calcium Channel Blocker; GSK-3ß = Glycogen synthase kinase 3ß; MasR = Mitochondrial Assembly Receptor; NO = nitric oxide; p-tau = phosphorylated tau.

Furthermore, higher bradykinin levels are linked to Aß deposition, and its presence may enhance B1R, accentuating amyloid toxicity. In addition, the Aß42-amyloid peptide can induce the plasma contact system and activate the kallikrein-kinin system (KKS) because of its negative charge [51,52,56]. As a result, an increase in bradykinin production takes place, enhancing cerebral inflammation and vascular permeability [50,51,52,53,54] and up-regulating bradykinin receptors again (Figure 4). 

On the contrary, there is evidence that ARBs reduce the Aß burden in mice models and can reduce p-tau and neurofibrillary tangles in the hippocampus [29]. Their neuroprotector effect is attributed, in part, to AT1R blockade while stimulating AT2R, AT4R, and MasR [63]. 

AT1R can release aldosterone and cause vasoconstriction, fluid retention, and the M1 phenotype of microglial cell activation, which releases pro-inflammatory cytokines [29,63]. In addition, AT1R is related to hypertension, heart dysfunction, brain ischemia, abnormal stress responses, BBB breakdown, and inflammation [64]. Thus, the AT1R/Ang II axis links to pro-inflammatory and prooxidant effects, increasing BBB permeability [3,29], as well as cognitive impairment and tau hyperphosphorylation through the activation of GSK3ß [14], which has an essential role in the modulation of insulin [7]. Moreover, microglial activation is higher in elderly patients, and Aß pathogenesis may exacerbate this process [3].

Conversely, AT2R causes angiogenesis, an NO increase, vasodilation, and the activation of the M2 phenotype of microglial cells, thereby releasing anti-inflammatory cytokines [29,63].

MasR produces anti-inflammatory, anti-oxidative, anti-fibrotic, vasodilation, and M2 activation effects [27,63], improving memory, learning, and long-period potentiation in mouse models [27]. Finally, insulin-regulated aminopeptidase is associated with vasodilation and long-term potentiation enhancement [3,28]. 

Interestingly, AT1R is expressed more in the brain than AT2R [29]. As a result, drugs acting as AT1R antagonists may promote AT2R, MasR, and insulin-regulated activation, which may become significant in cognitive abilities. Other described mechanisms of action by which ARBs may have a protector role in AD are neuronal differentiation, DNA repair, the modulation of the cerebral microvasculature, the reversion of oxidative stress and inflammation, and ischemic brain injury prevention [41].

This work shows a positive association between the tau/Aß42 ratio and the use of ACEi, with an opposite effect when compared to using ARBs in combination (Figure 4). These findings are broadly consistent with slower Aß [28,65] and tau progression when ARBs are consumed instead of ACEi. 

The previously described mechanisms may explain why we observed a higher t-tau/Aß42 ratio in patients taking ACEi and its contrary effect in patients taking ARBs. Compared to published work, metanalysis shows similar results in dementia risk prevention with ARBs consumption, whereas ACEi does not seem to reduce the risk of dementia [16,25] or reduce its risk less than ARBs [26]. In addition, the longitudinal study by Nation et el. in 2016 showed higher CSF Aß42 levels and lower p-tau levels over time when ARBs treated patients were compared to patients not taking antihypertensives. This study showed that Aß42 reduction was independent of age, the most influential risk factor of AD [41]. Finally, a metanalysis performed by D’Silva et al. in 2022 shows how other clinical trials in which ARBs were compared to a placebo obtained conflicting results. One trial showed less deterioration in episodic memory and attention, whereas others did not show differences. Moreover, when compared to CBB, cognitive improvements were not observed, but an increase in cerebral blood flow in several brain regions, including the parietal lobe, was observed [26].

Finally, it must be noted that in our study, ARBs showed a protective effect in combination with diuretics, which is the most prescribed combination. In this sense, several studies pointed out the possible role of diuretics in AD risk reduction [38,48]. Their possible role may be due to the effect of these drugs on reducing cerebrovascular events, such as silent vascular lesions, that are involved in white matter changes, a common hallmark in AD and other dementias [38]. In addition, diuretics may act as AD risk reducers by their vasorelaxant effect, which may counteract the vasoconstriction produced by amyloid pathogenesis [38,48]. Among them, thiazide diuretics and potassium-sparing diuretics stand out as AD risk reducers in the Cache County study [22]. Thus, the protective role of ARBs could be enhanced by the neuroprotector properties that diuretics seem to have.

### Strengths and Limitations

A strength of the present study is that the medications were registered according to exact dates and exact doses. In addition, the participants were classified as attending to CSF biomarkers levels, while most antihypertensive studies that correlate this medication with AD are based only on cognitive tests. In this sense, the present study provides an objective and accurate AD diagnosis. 

It should be considered that the inclusion criteria for this study were to consent to a lumbar puncture, which is an invasive intervention that dissuades potential participants, especially cognitively healthy adults. In spite of the required invasive sampling with some adverse side effects (headaches, pain), a relevant number of cognitively healthy participants was included in the present study. This is a strength because, to our knowledge, there are few studies about antihypertensives with a control sample based on CSF biomarkers, and published work has a few participants. On the contrary, this fact is also a limitation, and future studies including more participants are needed.

Lastly, it must be considered that the study was performed at an outpatient consultation center of the Cognitive Disorders Unit, where other healthcare professionals refer patients due to pathological suspicion or memory complaints. Moreover, adherence has not been verified, and genetic risk factors such as APOE e4 have not been analyzed.

## 5. Conclusions

High SBP, elderly age, and female gender are variables associated with a higher risk of AD diagnosis. In addition, calcium channel blockers and plain ACEi consumption are associated with a higher tau/Aß42 ratio, whereas consuming ARBs is associated with a lower tau/Aß42 ratio. Thus, ARBs should be considered a primary antihypertensive option for patients at risk of AD. 

## Figures and Tables

**Table 1:** Demographic and clinical variables for the participants’ groups.

Variable	AD (n = 223)	Control (n = 57)
**Age** (years, median (IQR))	71 (67.5, 74)	65 (62, 69)
**Sex** (female, n (%))	135 (60.54%)	25 (43.86%)
**CSF biomarkers**		
Aß42 levels (pg/mL, median (IQR))	600 (468.04, 702.1)	1206.15 (996, 1472)
t-tau levels (pg/mL, median (IQR))	586 (414, 837)	240 (182, 313)
p-tau levels (pg/mL, median (IQR))	92 (72.5, 131)	42 (32, 56)
Ratio t-tau/Aß42 (median (IQR))	0.94 (0.68, 1.43)	0.19 (0.14, 0.24)
**Smoking history**		
No (n, %)	145 (65.02%)	34 (59.65%)
No (Ex-smoker), (n, %)	45 (20.18%)	10 (17.54%)
Yes (n, %)	33 (14.8%)	13 (22.81%)
**Number of concomitant medications**	5 (3, 7)	3 (2, 5)
**Total cholesterol** (mg/dL, median (IQR))	189 (165.25, 212)	196 (170, 222)
**HDL cholesterol** (mg/dL, median (IQR))	55.5 (47, 66.25)	55 (44, 67.6)
**Lipid-modifying agents** (n, %)	123 (55.16%)	28 (49.12%)
**Antidiabetic drugs** (n, %)	36 (16.14%)	11 (19.3%)
**Systolic blood pressure** (mmHg, median (IQR))	135 (124.58, 143.42)	130 (118, 139)
**Diastolic blood pressure (DBP)** (mmHg, median (IQR))	75 (70, 81)	78 (71, 82.5)
**Hypertension** (n, %)	122 (54.71%)	30 (52.63%)

Aß42 = ß amyloid 42; CSF = cerebrospinal fluid; dL = deciliter; IQR = interquartile range; mg = milligrams; mL = milliliters; mmHg = millimeters of mercury; n = number of patients; pg = picograms; p-tau = phosphorylated tau; t-tau = total tau.

**Table 2:** Antihypertensive prescription for the participants’ groups.

Variable	AD (n = 223)	Control (n = 57)
**Antihypertensive drugs prescription** (n, %)	116 (52.02%)	29 (50.88%)
**Age at first prescribed antihypertensive treatment** (years, median (IQR))	61 (58, 65)	56 (53, 62)
**Years since 1st antihypertensive treatment prescription** (years, median (IQR))	7 (0, 11)	2 (0, 10)
**Number of antihypertensives daily intake** (n, %)		
0	107 (47.98%)	28 (49.12%)
1	59 (26.46%)	11 (19.3%)
2	40 (17.94%)	11 (19.3%)
3	16 (7.17%)	6 (10.53%)
4	1 (0.45%)	0 (0%)
5	0 (0%)	1 (1.75%)
**Doxazosin prescription** (n, %)	2 (0.9%)	2 (3.51%)
**Diuretics prescription** (n, %)	9 (4.04%)	3 (5.26%)
**Peripheral vasodilators** (n, %)	3 (1.35%)	1 (1.75%)
**Calcium dobesilate prescription** (n, %)	0 (0%)	1 (1.75%)
**Beta-blocking agents prescription** (n, %)	20 (8.97%)	1 (1.75%)
**Calcium channel blockers prescription** (n, %)	36 (16.14%)	8 (14.04%)
**Agents acting on the renin-angiotensin system** (n, %)	90 (40.36%)	25 (43.86%)

IQR = Interquartile range; n = number of patients.

**Table 3:** Elastic net model for CSF biomarkers (Aß-42, p-tau, t-tau, ratio t-tau/Aß42) from antihypertensive variables.

Variable	Aß42 Model (Estimate)	p-tau Model (Estimate)	t-tau Model (Estimate)	Ratio tau/Aß42 Amyloid Model (Estimate)
Sex (male)	-	-	−0.087	−0.1
Age	−0.015	0.015	0.024	0.047
Antidiabetic drugs	0.043	-	-	−0.035
Total cholesterol (mg/dL)	-	-	−0.001	−0.001
Diastolic blood pressure	-	-	−0.002	-
Number of antihypertensives	0	-	-	-
Doxazosin prescription	0.123	-	−0.107	−0.481
Calcium dobesilate prescription	0.175	-	-	−0.421
Calcium channel blockers	-	-	-	0.053
ACEi, plain	-	-	0.059	0.05
ARBs, combinations	-	-	−0.041	−0.206
lambda	0.067	0.123	0.069	0.075

Alpha = 0.5. ACEi = Angiotensin-converting enzyme inhibitors; ARBs = Angiotensin receptor blockers; Aß42 = ß amyloid 42; dL = deciliter; p-tau = phosphorylated tau; t-tau = total tau.

**Table 4:** Renin-angiotensin drugs subgroups consumption between AD and control patients.

Variable	AD	Control
**Angiotensin-converting enzyme inhibitors prescription** (n, (%))	40 (17.94)	5 (8.77)
**Angiotensin-converting enzyme inhibitors prescription duration** (months, median (IQR))	42 (11, 106)	19 (1, 48)
**Plain angiotensin-converting enzyme inhibitors prescription** prescription (n, (%))	30 (13.45)	1 (1.75)
**Combinations of angiotensin-converting enzyme inhibitors** prescription prescription (n, (%))	10 (4.48%)	4 (7.02%)
**Angiotensin-converting enzyme inhibitors prescription without medical records of angiotensin II receptor blockers prescription** (n, (%))	33 (14.8%)	4 (7.02%)
**Angiotensin II receptor blockers prescription** (n, (%))	50 (22.42%)	20 (35.09%)
**Angiotensin II receptor blockers duration** (months, median (IQR))	83 (56.75, 118.75)	69 (39, 95)
**Plain angiotensin II receptor blockers** (n, (%))	21 (9.42%)	7 (12.28%)
**Combinations of angiotensin II receptor blockers** (n, (%))	29 (13%)	12 (21.05%)
**Angiotensin II receptor blockers prescription without medical records of angiotensin-converting enzyme inhibitors prescription** (n, (%))	43 (19.28)	18 (31.58)

IQR = Interquartile range; n = number of patients.

## Data Availability

The data presented in this study are available on request from the corresponding author.

## Supplementary Materials

The following supporting information can be downloaded at https://www.mdpi.com/article/10.3390/pharmaceutics15030924/s1, Table S1: Multivariate logistic regression to obtain the risk of AD from SBP and DBP and controlled by age, sex and antihypertensive intake; Table S2: Association between t-tau/Aß42 ratio and sex, age, ARBs and ACEi drugs:ç; Table S3: Relationship between renin-angiotensin-system- acting agents and Alzheimer’s Disease biomarkers.
