# Supplementary material for: Angiotensin II Receptor Blockers Reduce Tau/Aß42 Ratio: A Cerebrospinal Fluid Biomarkers’ Case-Control Study
Source: Pharmaceutics. 2023 Mar 12;15(3):924. doi: 10.3390/pharmaceutics15030924 (PMC10059654; doi:10.3390/pharmaceutics15030924)
Supplement: Supplementary file 1 [file pharmaceutics-15-00924-s001.zip › pharmaceutics-2251269-supplementary.pdf]

Table S1. Multivariate logistic regression to obtain the risk of AD from SBP and DBP and controlled by age, sex and antihypertensive intake.

Variables	Estimate	Standard Error	OR	Lower 95%	Upper 95%	P-value
(Intercept)	-11.167	2.739	0	0	0.003	<0.001
Age	0.161	0.032	1.174	1.105	1.255	<0.001
Sex (male)	-0.667	0.368	0.513	0.246	1.051	0.07
SBP	0.035	0.016	1.036	1.004	1.071	0.033
DBP	-0.033	0.024	0.967	0.921	1.013	0.166
Antihypertensive treatment	-0.07	0.373	0.933	0.446	1.941	0.852

DBP= Diastolic blood pressure; OR= Odds ratio; SBP= Systolic blood pressure.

Table S2. Association between t-tau/Aß42 ratio and sex, age, ARBs and ACEi drugs.

Variables	Estimate	Standard error	Lower- 95	Upper- 95	P-value
(Intercept)	-3.989	0.593	-5.157	-2.821	<0.001
Age	0.055	0.009	0.038	0.072	<0.001
Sex (male)	-0.226	0.1	-0.423	-0.029	0.025
ARBs, combinations	-0.334	0.142	-0.613	-0.055	0.019
ACEi, plain	0.19	0.162	-0.128	0.509	0.24
R Squared	0.1662				
Adj. R Squared	0.1541				

ACEi: Angiotensin converting enzyme inhibitors; ARBs: Angiotensin receptor blockers.

Table S3. Relationship between renin-angiotensin-system- acting agents and Alzheimer's Disease biomarkers.

Step (Figure 4)	Relationship between renin-angiotensin-system (RAS)-acting agents and Alzheimer's Disease biomarkers	References
**1. ACEi**	Reduces Ang-II synthesis, enhancing acetylcholine and Ang-IV release	[1]
	Its chronic exposure may produce increased Aß42 levels in the brain	[2]
**2- BK**	Bradykinin receptors produce phospholipase C activation, which increases intracellular calcium mobilization, releases NO and prostaglandins and activate PKC, MAPK and NF-kB	[3-5]
**3. B1R**	Its expression is induced by MAPK, NF-kB and proinflammatory cytokines. Up-regulated in chronic inflammation	[4-11]

	Its expression and activation are linked to Aβ deposition and neuroinflammation in a mouse model of Alzheimer's disease	
**4. B2R**	B2R produces PKA as well, with which phospholipase C, phospholipase A2 and MAPK, can activate glutamate, NMDA and AMPA receptors, improving spatial memory. Up-regulated in acute inflammation.	[4,6,7,10]
**5. XIIa factor**	FXIIa activation of plasma prekallikrein leads to the release of bradykinin via cleavage of the intact form of HK	[4,12]
**6. ARBs**	Blocks AT1R, promoting the release of Ang II available to bind to Ang-IV, thereby facilitating memory and learning. It stimulates the PPARy, which is related to neuroprotection	[1]
	They are associated with a lower amyloid burden in non APOEe4+	[13]
**7. AT1**	It is naturally upregulated with ageing and its activation promotes M1 microglial phenotype activation, which releases TNF, IL-1ß, NO and ROS. It can impair cognitive function.	[14]
	Its activity is directly liked to inflammation, oxidation, neurotoxicity, and BBB damage	[15]
**8. AT2**	Linked to cell proliferation, differentiation, apoptosis, and regeneration of tissues	[16]
	Not detected on healthy microglia. Promotes M2 microglia phenotype, which produces anti-inflammatory cytokines	[14]
**9. MasR**	Neuroprotective effect. Enhances long-term potentiation	[14,16,17]
	Reduces oxidative stress: Increases NO and decreases mitochondrial respiration. Induces M2 microglia polarization, which enhances BDNF	[14]
**10. Ang IV**	Enhances cholinergic transmission and improves cognitive abilities.	[1,14,15]
**11. ACE2**	Its overexpression is associated with decreased activity of ACE1, Ang-II and AT1R expression, as well as decreased oxidative stress and neurofilament. It converts Aß43 into Aß42	[17]
**12. ACE1**	Increased ACE activity in patients with AD. Related to amyloid load and severity of AD	[2,17,18]
	It converts Aß42 into Aß40	[2,14,16,17]
**13. Doxazosin**	Doxazosin may reduce p-tau formation due to PI-3K/Akt-mediated inhibition of GSK-3 activity	[19]
**14. NO**	Neuroprotective at normal brain concentration. Its excess of its deficiency is associated with neurodegeneration and neuronal damage	[20]
**15. GSK3ß**	Angiotensin may activate GSK3ß and induce tau phosphorylation	[18]
	It may be activated by PS1 overexpression. Its overexpression is associated with disrupted islet ß cells and with tau hyperphosphorylation	[21]
**16. CCB**	Downregulate amyloid levels and slow its production	[22,23]
	Enhance cerebral vascularization	[24]

	Their effect on Aß clearance depends on their ability to facilitate Aß transcytosis. Drugs such as nimodipine or nitrendipine have this property	[25]

Aß= Amyloid beta peptide; ACEi= Angiotensin converting enzyme inhibitors; ACE1= Angiotensin converting enzyme 1; ACE2= Angiotensin converting enzyme 2; ARBs= Angiotensin receptor blockers; BDNF= brain-derived neurotrophic factor; BK= Bradykinin; AT1= Angiotensin 1 Receptor; AT2= Angiotensin 2 Receptor; B1R= Bradykinin 1 receptor; B2R= Bradykinin 2 receptor; CCB= Calcium channel blockers; GSK-3= Glycogen synthase kinase 3; HK= high molecular weight kininogen; MAPK= mitogen activated protein kinase; MasR= Mitochondrial Assembly Receptor; NF-kB= nuclear factor-kB; NO= nitric oxide; PKA= protein kinase A; PI-3K/Akt= Phosphatidylinositol 3-kinases/ protein kinase B; PKC= protein kinase isoforms; PPARy= peroxisome proliferator activated receptor gamma; ROS= Reactive Oxygen Species

References

1. Urmila, A.; Rashmi, P.; Nilam, G.; Subhash, B. Recent Advances in the Endogenous Brain Renin-Angiotensin System and Drugs Acting on It. *J. Renin Angiotensin Aldosterone Syst.* **2021**, *2021*, 9293553. https://doi.org/10.1155/2021/9293553.
2. Barus, R.; Béné, J.; Deguil, J.; Gautier, S.; Bordet, R. Drug interactions with dementia-related pathophysiological pathways worsen or prevent dementia. *Br. J. Pharmacol.* **2019**, *176*, 3413–3434. https://doi.org/10.1111/bph.14607.
3. Viel, A.T.; Buck, S.H. Kallikrein-kinin system mediated inflammation in Alzheimer's disease in vivo. *Curr. Alzheimer Res.* **2011**, *8*, 59–66. https://doi.org/10.2174/156720511794604570.
4. Dutra, R.C. Kinin receptors: Key regulators of autoimmunity. *Autoimmun. Rev.* **2017**, *16*, 192–207. https://doi.org/10.1016/j.autrev.2016.12.011.
5. Zhang, X.; Yu, R.; Wang, H.; Zheng, R. Effects of rivastigmine hydrogen tartrate and donepezil hydrochloride on the cognitive function and mental behavior of patients with Alzheimer's disease. *Exp. Ther. Med.* **2020**, *20*, 1789–1795. https://doi.org/10.3892/etm.2020.8872.
6. Mugisho, O.O.; Robilliard, L.D.; Nicholson, L.F.B.; Graham, E.S.; O'Carroll, S.J. Bradykinin receptor-1 activation induces inflammation and increases the permeability of human brain microvascular endothelial cells. *Cell Biol. Int.* **2019**, *44*, 343–351. https://doi.org/10.1002/cbin.11232.
7. Nunes, M.A.; Toricelli, M.; Schöwe, N.M.; Malerba, H.N.; Dong-Creste, K.E.; Farah, D.M.; De Angelis, K.; Irigoyen, M.C.; Gobeil, F.; Araujo Viel, T.; et al. Kinin B2 Receptor Activation Prevents the Evolution of Alzheimer's Disease Pathological Characteristics in a Transgenic Mouse Model. *Pharmaceuticals* **2020**, *13*, 288. https://doi.org/10.3390/ph13100288.
8. Singh, P.K.; Chen, Z.L.; Strickland, S.; Norris, E.H. Increased Contact System Activation in Mild Cognitive Impairment Patients with Impaired Short-Term Memory. *J. Alzheimers Dis.* **2020**, *77*, 59–65. https://doi.org/10.3233/jad-200343.
9. Bitencourt, R.M.; de Souza, A.C.; Bicca, M.A.; Pamplona, F.A.; de Mello, N.; Passos, G.F.; Medeiros, R.; Takahashi, R.N.; Calixto, J.B.; Prediger, R.D. Blockade of hippocampal bradykinin B1 receptors improves spatial learning and memory deficits in middle-aged rats. *Behav. Brain Res.* **2017**, *316*, 74–81. https://doi.org/10.1016/j.bbr.2016.08.041.
10. Ji, B.; Wang, Q.; Xue, Q.; Li, W.; Li, X.; Wu, Y. The Dual Role of Kinin/Kinin Receptors System in Alzheimer's Disease. *Front. Mol. Neurosci.* **2019**, *12*, 234. https://doi.org/10.3389/fnmol.2019.00234.
11. Singh, P.K.; Chen, Z.L.; Ghosh, D.; Strickland, S.; Norris, E.H. Increased plasma bradykinin level is associated with cognitive impairment in Alzheimer's patients. *Neurobiol. Dis.* **2020**, *139*, 104833. https://doi.org/10.1016/j.nbd.2020.104833.
12. Chen ZL, Singh P, Wong J, Horn K, Strickland S, Norris EH. An antibody against HK blocks Alzheimer's disease peptide β-amyloid-induced bradykinin release in human plasma. Proc Natl Acad Sci U S A 2019;116:22921–3. https://doi.org/10.1073/PNAS.1914831116.
13. Ouk M, Wu CY, Rabin JS, Edwards JD, Ramirez J, Masellis M, et al. Associations between brain amyloid accumulation and the use of angiotensin-converting enzyme inhibitors versus angiotensin receptor blockers. Neurobiol Aging 2021;100:22–31. https://doi.org/10.1016/J.NEUROBIOLAGING.2020.12.011.

14. Ribeiro VT, de Souza LC, Simões e Silva AC. Renin-Angiotensin System and Alzheimer's Disease Pathophysiology: From the Potential Interactions to Therapeutic Perspectives. Protein Pept Lett 2020;27:484–511. https://doi.org/10.2174/0929866527666191230103739.
15. Gouveia F, Camins A, Ettcheto M, Bicker J, Falcão A, Cruz MT, et al. Targeting brain Renin-Angiotensin System for the prevention and treatment of Alzheimer's disease: Past, present and future. Ageing Res Rev 2022;77:101612. https://doi.org/10.1016/J.ARR.2022.101612.
16. Naffah-Mazzacoratti M da G, Gouveia TLF, Simões PSR, Perosa SR. What have we learned about the kallikrein-kinin and renin-angiotensin systems in neurological disorders? World J Biol Chem 2014;5:130. https://doi.org/10.4331/WJBC.V5.I2.130.
17. Evans CE, Miners JS, Piva G, Willis CL, Heard DM, Kidd EJ, et al. ACE2 activation protects against cognitive decline and reduces amyloid pathology in the Tg2576 mouse model of Alzheimer's disease. Acta Neuropathol 2020;139:485. https://doi.org/10.1007/S00401-019-02098-6.
18. Wharton W, Zhao L, Steenland K, Goldstein FC, Schneider JA, Barnes LL, et al. Neurofibrillary Tangles and Conversion to Mild Cognitive Impairment with Certain Antihypertensives. J Alzheimers Dis 2019;70:153–61. https://doi.org/10.3233/JAD-190011.
19. Coelho BP, Gaelzer MM, dos Santos Petry F, Hoppe JB, Trindade VMT, Salbego CG, et al. Dual Effect of Doxazosin: Anticancer Activity on SH-SY5Y Neuroblastoma Cells and Neuroprotection on an In Vitro Model of Alzheimer's Disease. Neuroscience 2019;404:314–25. https://doi.org/10.1016/J.NEUROSCIENCE.2019.02.005.
20. Kumar Nath A, Ghosh Dey S. Dalton Transactions FRONTIER Simultaneous binding of heme and Cu with amyloid β peptides: active site and reactivities 2022. https://doi.org/10.1039/d2dt00162d.
21. Zhang Y, Huang N qu, Yan F, Jin H, Zhou S yu, Shi J shan, et al. Diabetes mellitus and Alzheimer's disease: GSK-3β as a potential link. Behav Brain Res 2018;339:57–65. https://doi.org/10.1016/J.BBR.2017.11.015.
22. Den Brok MGHE, Willem Van Dalen J, Abdulrahman H, Larson EB, Van Middelaar T, Van Gool WA, et al. Antihypertensive Medication Classes and the Risk of Dementia: A Systematic Review and Network Meta-Analysis 2021. https://doi.org/10.1016/j.jamda.2020.12.019.
23. Hwang D, Kim S, Choi H, Oh IH, Kim BS, Choi HR, et al. Calcium-Channel Blockers and Dementia Risk in Older Adults – National Health Insurance Service – Senior Cohort (2002–2013) –. Circ J 2016;80:2336–42. https://doi.org/10.1253/CIRCJ.CJ-16-0692.
24. Lebouvier T, Chen Y, Duriez P, Pasquier F, Bordet R. Antihypertensive agents in Alzheimer's disease: beyond vascular protection. Expert Rev Neurother 2020;20:175–87. https://doi.org/10.1080/14737175.2020.1708195.
25. Chuang YF, Breitner JCS, Chiu YL, Khachaturian A, Hayden K, Corcoran C, et al. Use of diuretics is associated with reduced risk of Alzheimer's disease: the Cache County Study. Neurobiol Aging 2014;35:2429–35. https://doi.org/10.1016/J.NEUROBIOLAGING.2014.05.002.
